# Short-Term Morphological Response of Polypropylene Membranes to Hypersaline Lithium Fluoride Solutions: A Multiscale Modeling Approach

**DOI:** 10.3390/ijms26157380

**Published:** 2025-07-30

**Authors:** Giuseppe Prenesti, Pierfrancesco Perri, Alessia Anoja, Agostino Lauria, Carmen Rizzuto, Alfredo Cassano, Elena Tocci, Alessio Caravella

**Affiliations:** 1 Department of Computer Engineering, Modelling, Electronics and System Engineering (DIMES), University of Calabria, Via P. Bucci 42C, 87036 Rende, CS, Italy; njalss01e50c352d@studenti.unical.it; 2 Institute on Membrane Technology–National Research Council of Italy (CNR-ITM), Via P. Bucci 17C, 87036 Rende, CS, Italy; c.rizzuto@itm.cnr.it (C.R.); a.cassano@itm.cnr.it (A.C.); e.tocci@itm.cnr.it (E.T.); 3 Department of Civil, Chemical, Environmental and Materials Engineering (DICAM), Alma Mater Studiorum University of Bologna, Via Zamboni 33, 40126 Bologna, BO, Italy; perripierfrancesco96@gmail.com; 4 Department of Engineering for Innovation, University of Salento (UNISALENTO), Corpo Z, Campus Ecotekne, SP.6 per Monteroni, 73047 Lecce, LE, Italy; agostino.lauria@unisalento.it

**Keywords:** membrane-assisted crystallization, membrane morphology, computational fluid dynamics simulations, molecular dynamics simulations

## Abstract

Understanding the early-stage physical interactions between polymeric membranes and supersaturated salt solutions is crucial for advancing membrane-assisted crystallization (MCr) processes. In this study, we employed molecular dynamics (MD) simulations to investigate the short-term morphological response of an isotactic polypropylene (PP) membrane in contact with LiF solutions at different concentrations (5.8 M and 8.9 M) and temperatures (300–353 K), across multiple time points (0, 150, and 300 ns). These data were used as input for computational fluid dynamics (CFD) analysis to evaluate structural descriptors of the membrane, including tortuosity, connectivity, void fraction, anisotropy, and deviatoric anisotropy, under varying thermodynamic conditions. The results show subtle but consistent rearrangements of polymer chains upon exposure to the hypersaline environment, with a marked reduction in anisotropy and connectivity, indicating a more compact and isotropic local structure. Surface charge density analyses further suggest a temperature- and concentration-dependent modulation of chain mobility and terminal group orientation at the membrane–solution interface. Despite localized rearrangements, the membrane consistently maintains a net negative surface charge. This electrostatic feature may influence ion–membrane interactions during the crystallization process. While these non-reactive, short-timescale simulations do not capture long-term degradation or fouling mechanisms, they provide mechanistic insight into the initial physical response of PP membranes under MCr-relevant conditions. This study lays a computational foundation for future investigations bridging atomistic modeling and membrane performance in real-world applications.

## 1. Introduction

Membrane crystallization (MCr) is emerging as a promising and environmentally sustainable technology for recovering high-value compounds. This innovative approach combines membrane technology with the crystallization process [1]. Specifically, MCr follows the membrane distillation step, which is used to recover fresh water and concentrate the feed solution until it reaches saturation [1]. In a membrane crystallizer, a solution containing a non-volatile solute, such as brines, lithium salt-enriched solutions, or mother liquors resulting from separation processes, is brought into contact with the distillate side through a hydrophobic microporous membrane [2]. The membrane prevents mass transfer in the liquid phase, allowing only vapor-phase transfer driven by a vapor pressure gradient [3]. As a result, the continuous evaporation of the solvent leads to the supersaturation of the solute, facilitating crystallization with the aid of the membrane [4,5,6]. The driving force for this process is the transmembrane vapor pressure gradient, which can be induced through the application of a thermal gradient in thermal membrane crystallizers or by a concentration gradient in osmotic membrane crystallizers [7,8]

This method is attracting increasing interest, since numerous studies have shown that it offers well-controlled nucleation and growth kinetics, high crystallization rates, and reduced induction times. Additionally, it possesses potential advantages in terms of crystal quality and energy efficiency, as it can operate at relatively low temperatures and utilize alternative heat sources [1,9,10,11].

A supersaturated solution that undergoes crystallization can be viewed as a dynamic, multi-component system where many solute molecules move within a lattice of solvent molecules. Collisions between solute molecules may initiate the formation of self-assembly clusters and pre-nucleation aggregates. However, transitioning to stable cluster formation is thermodynamically constrained by an energetic barrier that must be overcome. In this context, the membrane surpasses its role as a mere physical barrier separating two distinct phases; it intervenes by reducing the Gibbs free energy required for the formation of stable critical nuclei, thereby increasing the probability of nucleation in its vicinity compared to other areas (heterogeneous nucleation) [7].

In recent years, MCr has been explored as a promising method for lithium recovery from brines, owing to the growing interest in lithium for energy storage applications. Lithium is an essential element in the production of lithium-ion batteries (LiBs), which are crucial for a wide range of electronic devices and electric vehicles due to their high energy density, power output, and long cycle life. The lithium-ion battery market is rapidly expanding, driven by the increasing commitment to promote sustainable mobility through the electrification of transportation. Projections suggest that, by 2030, lithium supply may fall short of global demand.

Thus, the need for lithium recovery arises from a combination of increasing technological relevance, limited availability of primary resources, and significant environmental and economic advantages. Specifically, recycling spent batteries presents a valuable commercial opportunity, as producing cathode materials (including lithium, nickel, cobalt, and manganese) constitutes a significant portion of the total cost of manufacturing an LiB cell [12].

The growing demand for lithium, along with climate change and environmental concerns, has emphasized the need for more sustainable processes, such as MCr, in lithium-ion battery recycling. This poses stringent requirements on membrane performance. Specifically, membranes must operate under high salinity, temperature, and pH without undergoing fouling, wetting, or morphological degradation.

The performance of MCr is critically dependent on selected operating conditions, including temperature, pressure, the concentration and nature of the precipitating species, and most importantly, the physical and morphological characteristics of the membrane barrier [7,13]. For example, temperature significantly impacts both the vapor pressure gradient across the membrane, which is the driving force for the process, and the solubility of the components in the solution. Feed flow rates affect the system’s hydrodynamics and mixing efficiency, while the concentration level (or degree of saturation) directly influences crystal yield. Higher levels of supersaturation can enhance nucleation rates and contribute to improved crystal purity [14].

Recently, Jiang et al. [15] investigated the engineering and effective control of the MCr process, conceptualizing membranes used in this process as microscale interfacial technology. The study demonstrated how various morphological attributes—such as porosity, pore size distribution, the orientation of polymer chains, surface contact angle, roughness, and additional parameters—can significantly influence the overall performance of the separation process. By modulating these properties, it is possible to ensure a uniform degree of micromixing within the interfacial liquid layer, adjust the contact area, enhance nucleation activity, improve selectivity, accelerate crystal growth kinetics, and influence the permeability of the liquid phase in the membrane structure.

To enhance durability and performance, bioinspired and surface-engineered membranes have been developed. For instance, Jiang et al. [16], developed bioinspired composite membranes with a hybrid micro- and nanostructure to be used in membrane distillation of high-salinity water. They demonstrated the feasibility of modulating the morphological properties of polymeric membranes to prevent salt crystal deposition during membrane distillation processes. Specifically, commercial polypropylene (PP) membranes underwent various treatments, including plasma treatment, to increase pore size and introduce active graft-specific groups, loading with SiO_2_ nanoparticles, and fluorination to enhance their hydrophobicity. The combination of these treatments and morphological modifications resulted in a membrane with a complex surface morphology and exceptional hydrophobicity (with a water contact angle of 160.59°), effectively inhibiting the wetting of the porous surface, counteracting salt crystal deposition, and significantly reducing fouling. In another approach, Al-Shaeli et al. [17] focused on inorganic layered polymer membranes and surface engineering strategies. One notable modification involved the incorporation of zinc oxide (ZnO) nanorods along with the application of a perfluorodecyltriethoxysilane (PDTS) coating, which produced membranes with outstanding anti-fouling and anti-wetting properties. Likewise, membranes modified with titanium dioxide (TiO_2_) nanorods achieved salt rejection rates exceeding 99.9% in membrane distillation applications for treating high-salinity solutions. In contrast, untreated pristine membranes displayed a gradual decline in separation performance due to pore wetting and foreshortening during operation. Another modification strategy involved incorporating halloysite nanotubes (m-HNTs) [17], which can be deposited on porous polyacrylonitrile (PAN) membranes due to their hollow tubular structure formed by rolled aluminosilicate sheets. The presence of a dense, well-aligned, and oriented layer increased the surface area and improved the mechanical stability of the membrane. Furthermore, the hollow structure of these nanotubes, with negative surface charges, provided a high level of selectivity for molecular transport.

The study conducted by Li et al. [1] showed that the hydrophobicity of a membrane, a crucial feature in both membrane distillation and MCr, is primarily influenced by its surface energy and roughness. A reduction in surface energy leads to lower affinity between the membrane and the liquid phase, resulting in increased hydrophobicity. Similarly, as described by the Cassie–Baxter equation, an increase in the surface roughness of the material expands the gas–liquid contact area and enhances the interfacial repulsion effect towards the liquid phase.

One surface modification strategy involves grafting sulfobetaine methacrylate (SBMA) onto a hydrophobic polyvinylidene fluoride (PVDF) membrane. This modification imparts anti-fouling and anti-wetting properties to the membrane, yielding a slightly lower contact angle while achieving a higher transmembrane flux than the unmodified membrane. Other examples of surface grafting utilize materials such as perfluoroalkylsilane (PFAS), fluoroalkylsilane (FAS), polydimethylsiloxane (PDMS), polyethylene glycol (PEG), and ethyl acrylate (EA). These examples highlight the versatility of surface modification strategies in adjusting membrane properties.

Additionally, the study explores blending fluorinated polymers with inorganic nanoparticles, including titanium dioxide (TiO_2_), silicon dioxide (SiO_2_), and calcium carbonate (CaCO_3_). This approach involves incorporating modifying materials into the casting solutions, resulting in membranes with enhanced hydrophobicity, improved mechanical strength, and better anti-fouling capabilities. Such modifications directly and significantly influence membrane performance in membrane distillation and MCr processes [1].

Perrotta et al. [18] also demonstrated that different crystalline phases of PVDF—amorphous, α, and β forms—exhibit distinct morphological characteristics that significantly affect the nucleation and growth behavior of sodium chloride (NaCl) crystals during membrane crystallization. Their study shows that amorphous PVDF (density 1.7 ± 0.015 g/cm^3^) promotes a higher nucleation rate, leading to shorter induction times (0 ns) and the formation of a greater number of smaller yet more regular clusters compared to the α and β forms (densities of 1.925 g/cm^3^ and 1.931 g/cm^3^, respectively). This behavior is attributed to the polymer chains’ morphological characteristics: amorphous PVDF has a free volume fraction of approximately 16.54% between the chains (compared to 2.3 Å for the β phase and 2.2 Å for the α phase), which allows water molecules to move more freely between them. This mobility facilitates the adsorption of water onto the surface and promotes more rapid supersaturation of the feed solution. The water uptake for the amorphous phase reaches significantly higher values (2.55 ± 0.55%) compared to the α and β phases, which have more compact and tightly interconnected crystalline structures that hinder water molecule mobility.

Further investigation into the nucleation and growth of NaCl crystals via MCr was conducted using PVDF membranes functionalized with graphene (at 0.5%, 5%, and 10% weight percentages of graphene nanoparticles) [19]. The incorporation of graphene induces morphological changes in the membrane structure, promoting the formation of cavities between PVDF chains and graphene flakes. These void regions allow for the infiltration of water molecules. The study demonstrates that graphene functionalization enhances the hydrophobicity of the membrane, reduces crystal nucleation time by up to 190%, increases water removal from the feed solution, and shortens the distance between ionic species within the resulting crystalline aggregates [19].

Regarding the influence of membrane morphology on nucleation and crystal growth, another promising approach involves functionalizing membranes with plasmonic or photothermal materials. This increasingly common procedure in membrane technologies leads to morphological changes that can significantly alter membrane performance in relevant applications. The primary goal of this type of functionalization is to elevate the temperature at the membrane–feed interface through light irradiation, thereby reducing or eliminating thermal polarization (TP) and enhancing the driving force for mass transfer. However, integration processes, such as the homogeneous dispersion of nanoparticles, incorporation of nanofibers, and surface coatings, can induce morphological modifications that affect key properties like wettability, porosity, surface roughness, and flow resistance. These factors are critical in membrane distillation and MCr operations [20]. Utilizing intrinsically hydrophilic materials like polydopamine (PDA) while incorporating nanoparticles to achieve greater surface roughness can enhance membrane hydrophilicity. However, introducing nanomaterials such as silver nanoparticles (Ag NPs), MXenes, titanium nitride (TiN), antimony tin oxide (ATO), and magnetite (Fe_3_O_4_) may introduce additional resistance to mass flow, with reductions of up to 13%, despite the photothermal benefits [20,21]. A comprehensive analysis of these properties is essential in understanding how the morphological characteristics of the employed membranes influence key phenomena such as supersaturation control, nucleation kinetics, and crystal growth dynamics [22].

In a previous study, Prenesti et al. [22] selected polypropylene (PP) membranes for MCr operations involving lithium salts. This choice was primarily due to their highly hydrophobic nature, which promotes crystal nucleation and growth within the membrane pores, thereby enhancing process efficiency. Recent studies on the MCr of NaCl salts have shown that the morphology of PP membranes, characterized by a sponge-like internal structure and cavity-like features on the outer surface, favors the formation of anchoring sites where crystals can grow and adhere [10,23].

Quist-Jensen et al. [24] utilized commercially available PP membrane modules to conduct a comparative analysis of various MCr and MD configurations aimed at recovering lithium chloride (LiCl) from highly concentrated aqueous solutions. Their findings indicated that using PP membranes in vapor membrane distillation (VMD) allows for the treatment of solutions up to saturation levels, thereby facilitating the potential for crystallization.

Nevertheless, the influence of concentrated ionic environments on the structural response of polymeric membranes remains poorly understood at the molecular scale. While experimental studies provide valuable performance indicators, they cannot fully elucidate how polymer chains rearrange upon exposure to hypersaline solution, especially during the early stages of contact. This gap is particularly relevant when analyzing local morphological descriptors such as anisotropy, tortuosity, and surface charge distribution, which may respond to ionic strength and temperature variations prior to the onset of macroscopic degradation.

This study addresses this knowledge gap by combining atomistic molecular dynamics (MD) simulations with computational fluid dynamics (CFD) to investigate the short-term morphological response of isotactic polypropylene (PP) membranes in contact with LiF solutions. The work focuses on the nanoscopic evolution of key structural descriptors (tortuosity, connectivity, void fraction, anisotropy, and surface charge) under hypersaline conditions (5.8 M and 8.9 M) and across a temperature range (300–353 K).

In particular, the effective diffusivity tensor and the corresponding diffusional tortuosity tensor of the polymer matrix are computed using a methodology recently developed by Caravella et al. [25], based on a CFD approach implemented within the OpenFOAM^®^ (https://www.openfoam.com/) software suite. The membrane morphologies subjected to CFD analysis were extracted from MD trajectories at selected simulation times.

The MD simulations were carried out under a set of clearly defined assumptions [22]:

1. The membrane is modeled as a dense, non-porous, and non-reactive polymeric matrix, with no flux, porosity, or degradation pathways considered. This idealized setup allows for isolating local physical rearrangements of polymer chains in response to ionic strength and thermal stimuli.

2. Micropores and the distillation step are deliberately excluded from the model. The simulations focus exclusively on crystallization dynamics in a static, supersaturated solution, where salt concentration remains constant throughout. All morphological descriptors refer to spacing and topological reorganization among polymer chains, and do not account for actual porosity or long-term degradation phenomena. Moreover, no transport through pores or reactive aging processes is considered.

3. Simulations start from a pre-defined supersaturated state, enabling the direct observation of crystallization behavior modulated by the presence of the polymeric membrane.

This approach enables us to selectively explore how local interactions between the dense polymer matrix and the surrounding ionic solution drive morphological changes. By excluding mass flux and chemical degradation, the analysis focuses solely on short-term, interaction-driven phenomena. It is important to emphasize that the conclusions drawn in this study are strictly limited to the modeled conditions and should not be extrapolated to long-term performance scenarios.

Overall, the aim of this work is to establish a foundational understanding of how polymeric membranes initially respond—at the nanoscopic scale—to elevated salinity and temperature. These insights are intended to support the rational design of membranes and modeling frameworks for next-generation membrane-assisted crystallization processes.

## 2. Results and Discussion

### 2.1. Morphological Properties

It is experimentally established that membranes employed in MCr applications are susceptible to various structural alteration phenomena. These changes arise from the synergistic action of the feed solution’s temperature, the degree of oversaturation, and the heterogeneous nucleation and growth of crystals on the membrane surface [26,27]. Such factors can also induce a decrease in the polymer’s crystallinity and a rearrangement of the membrane polymer chains. Consequently, even highly thermally and chemically stable polymeric structures, such as polypropylene, may undergo significant modification. This confirms the critical importance of predicting and quantifying eventual membrane modifications.

To assess these effects, we investigated the morphological response of an idealized dense PP membrane model under hypersaline LiF solutions (5.8 M and 8.9 M) at three temperatures (300, 328, 353 K) and at three time points (0, 150, 300 ns).

In all analyses, the pristine (virgin) PP membrane, i.e., the membrane model prior to any exposure to the ionic solution, was used as a reference control.

By computing morphological descriptors such as tortuosity, connectivity, void fraction, and anisotropy across simulation time and conditions, and comparing them directly to the control membrane, we were able to assess whether and to what extent the membrane underwent structural rearrangements or maintained morphological integrity. These parameters allowed us to quantify changes in local chain arrangement and void topology, excluding any contribution from actual porosity or reactive degradation.

In all cases, simulations started from a pre-equilibrated and oversaturated LiF solution. Because the total ion concentration remains constant during the simulation and crystallization begins immediately, the time evolution of the membrane’s morphology reflects the early stages of nucleation and growth. Once the number of nuclei stabilizes (as confirmed in previous work [22]), no further morphological changes are expected. While longer simulations may be useful to investigate late-stage degradation or aging effects, the 300 ns time frame used in this study was sufficient for characterizing early membrane perturbation, which represents the most critical regime in membrane-assisted crystallization. These findings make the presented observations broadly generalizable to realistic MCr operating conditions involving highly supersaturated environments.

**Figure 1:**
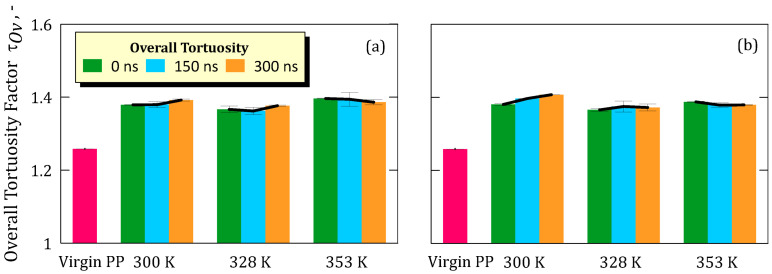
(**a**) Tortuosity with standard deviation of the membrane taken from simulation at salt concentration of 5.8 M; (**b**) Tortuosity with standard deviation of the membrane taken from simulation at salt concentration of 8.9 M.

The normalized overall tortuosity factor (τ_OV_) of the virgin membrane was 1.25. In the simulations at 5.8 M concentration, for the simulated temperatures of 300 K, 328 K, and 353 K, the values increased modestly to 1.38, 1.36, and 1.39, respectively (Figure 1a). Similarly, in the simulations at 8.9 M, the values recorded for the same temperatures were 1.39, 1.37, and 1.38, respectively (Figure 1b).

**Figure 2:**
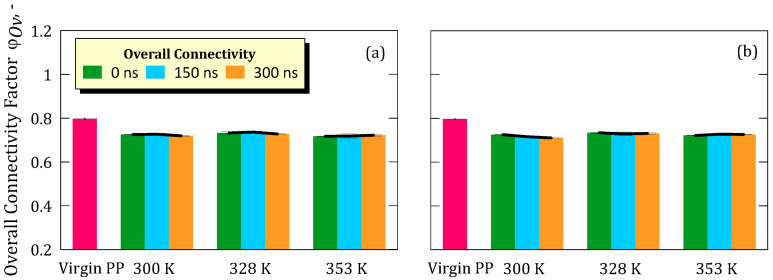
(**a**) Overall connectivity with standard deviation of the membrane taken from simulation at salt concentration of 5.8 M; (**b**) Overall connectivity with standard deviation of the membrane taken from simulation at salt concentration of M.

For the virgin PP membrane, the normalized overall connectivity factor (φ_Ov_) was 0.79. At the same time, for the simulations at 5.8 M and 300 K, 328 K, and 353 K, the values were 0.72, 0.73, and 0.71, respectively (Figure 2a). Instead, in the simulations at 8.9 M, values of 0.71, 0.73, and 0.72 were observed for the respective temperatures (Figure 2b).

**Figure 3:**
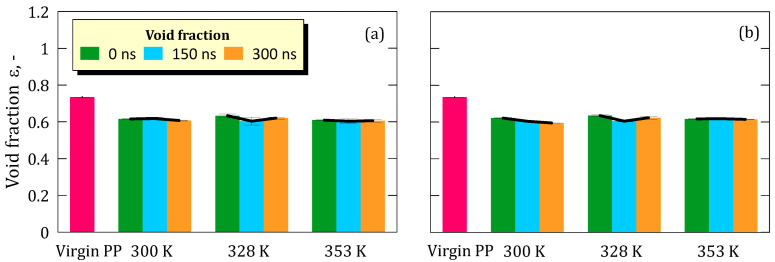
(**a**) Void fraction with standard deviation of the membrane taken from simulation at salt concentration of; (**b**) Void fraction with standard deviation of the membrane taken from simulation at salt concentration of 8.9 M.

The void fraction, like tortuosity and connectivity, remained constant over time (Figure 3). However, it decreased immediately when the virgin PP came into contact with the ionic solution. This indicates that the membrane maintains its hydrophobic nature, reducing inter-chain voids and thus limiting water uptake and adsorption.

No significant variations were observed over time for either concentration (5.8 M and 8.9 M) at all the investigated temperatures (Figure 3a,b). The invariance of key morphological descriptors of the polymeric matrix, i.e., tortuosity, connectivity, and void fraction, indicates that the polymer chain packing remains essentially unchanged throughout the simulation. These results are noteworthy, given that elevated temperature and ionic strength could be expected to disrupt polymer packing.

Simulating different temperature conditions (300, 328, and 353 K) serves to mimic varying feed temperatures. The literature evidence demonstrates that increasing the temperature of the feed solution is a particularly favorable condition during membrane crystallization operations, primarily due to an increase in the vapor pressure of the solution [27,28]. Therefore, the absence of incipient morphological changes at different solution temperatures reinforces the membrane’s suitability for such operational scenarios.

Moreover, in membrane crystallization, the local attainment of very high supersaturation concentrations, relative to the bulk, favors the formation of a supersaturation boundary layer. This facilitates interactions between the membrane surface and solute molecules, promoting the proper nucleation of crystals [13]. No differentiated morphological changes were found between the two distinct simulated supersaturation conditions; this confirms that the PP membrane, known for its versatility and wide use in these applications, can be successfully employed.

It is essential to note that the limited variation observed in key morphological descriptors, such as tortuosity and connectivity, which are intrinsically interdependent, does not imply that the membrane remains entirely unaffected by the hypersaline environment. Rather, our analysis reveals that short-term exposure induces localized rearrangements within the polymeric matrix. However, these changes do not alter the overall morphological architecture, which remains structurally stable under the tested temperature and concentration conditions. This indicates that, despite some degree of reorganization, the membrane maintains its nanostructural integrity during the early stages of interaction with the supersaturated solution.

For longer simulation times [22], there is usually a percentage reduction in membrane volume, accompanied by an increase in tortuosity and, consequently, a decrease in connectivity. These changes, although slight, indicate a rearrangement of the hydrophobic chains of the PP, which compact in contact with the ionic solution to minimize the absorption of water from the solution.

**Figure 4:**
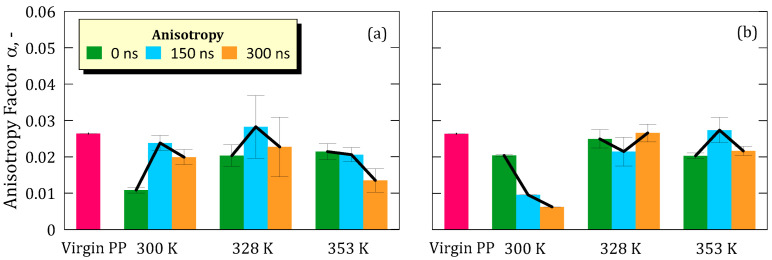
(**a**) Anisotropy with standard deviation of the membrane taken from simulation at salt concentration of 5.8 M; (**b**) Anisotropy with standard deviation of the membrane taken from simulation at salt concentration of 8.9 M.

**Figure 5:**
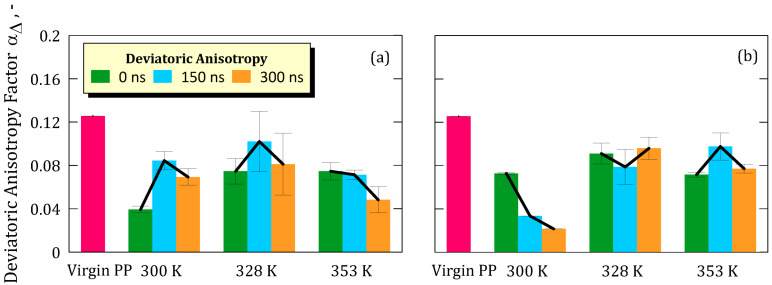
(**a**) Deviatoric anisotropy with standard deviation of the membrane taken from simulation at salt concentration of 5.8 M; (**b**) Deviatoric anisotropy with standard deviation of the membrane taken from simulation at salt concentration of 8.9 M.

The most significant changes were observed in anisotropy (Figure 4) and deviatoric anisotropy (Figure 5), for which a common pattern can be defined across all simulations.

The pristine PP membrane showed values of anisotropy (α) and deviatoric anisotropy (α_Δ_) of 0.026 and 0.125, respectively, which can be reasonably attributed to the random orientation of the polymer chains within the simulated cell.

Upon equilibration, under specific pressure (P) and temperature (T) conditions, both α and α_Δ_ values dropped markedly, suggesting a shift toward more isotropic chain configurations. The extent of change varied with temperature and concentration, with higher temperatures generally mitigating loss of anisotropy.

This initial decrease is attributed to the membrane–ion solution assembly procedure, where the overall system undergoes minimization and equilibration under NPT conditions, allowing the solution and membrane to converge and eliminate the initial empty space [22].

For a concentration of 5.8 M, we observed a decrease of 58.71% at 300 K, 23.11% at 328 K, and 18.94% at 353 K (Figure 4a).

At a concentration of 8.9 M, the percentage decreases were 22.73% (300 K), 5.30% (328 K) and 23.11% (353 K) (Figure 4b).

It is interesting to note how the extent of these changes tends to decrease with increasing simulated temperature, suggesting a less drastic interaction between the solution and the polymer structure as the temperature rises.

While anisotropy was partially recovered by 300 ns, the values remained consistently lower than those of the pristine membrane. This suggests a modest yet irreversible compaction and reorientation of the polymer chains at the interface. Such behavior is consistent with trends observed in earlier simulations [22] and with experimental evidence of local rearrangements in hydrophobic polymer matrices.

### 2.2. Electrostatic Charge Distribution

Surface charge distribution was also evaluated to determine whether local rearrangements influence electrostatic interactions.

**Figure 6:**
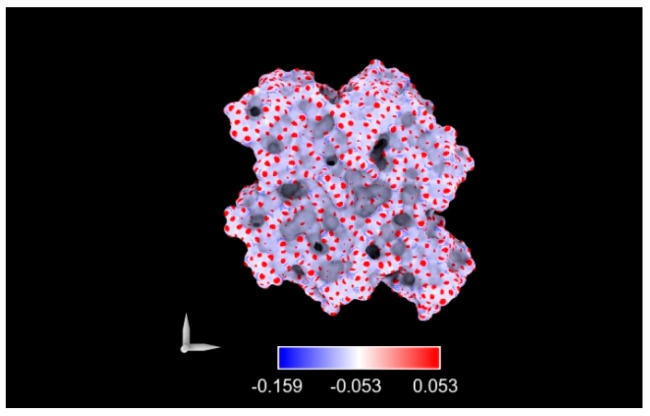
Charge distribution in virgin PP.

In its pristine state, the PP membrane exhibited a slight negative surface potential, consistent with zeta potential values reported in the literature (~−20 mV) [29] (Figure 6).

Figure 6 shows that the virgin PP had a slight negative superficial charge, meaning that central carbon atoms are less exposed towards the surface.

**Figure 7:**
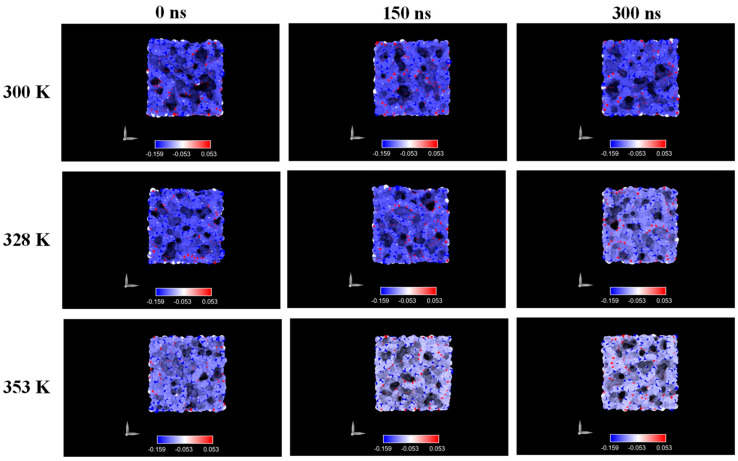
Charge distribution in PP membrane at different temperature conditions and 5.8 M.

Figure 7 illustrates the evolution of surface charge over time at various simulated temperatures for the system at 5.8 M LiF concentration. As soon as the membrane came into contact with the ionic solution, polymeric chains rearranged, resulting in a more negative superficial charge distribution. At the lowest temperature, the special charge was almost the same (Figure 7) in time. However, as temperature increases, terminal atoms of the polymer chains (specifically C1 and C2) tend to migrate toward the surface, resulting in a less-negative surface charge. This behavior is related to increased kinetic energy at higher temperatures, which enhances chain mobility and facilitates reorganization.

**Figure 8:**
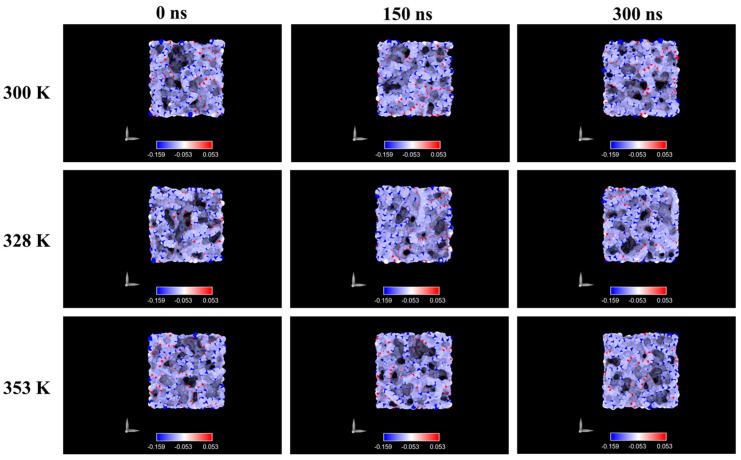
Charge distribution in PP membrane at different temperature conditions and 8.9 M.

At 8.9 M, however, the surface charge remained relatively stable over time and across temperatures (Figure 8). We attribute this to stronger Coulombic interactions in the higher-ionic-strength environment, which restricts chain mobility and prevents major reorganization.

The surface charge remained negative, although slightly less so compared to the 5.8 M system.

Across all conditions, the membrane maintained a net negative surface potential. These electrostatic characteristics, though modest, may influence ion–membrane interactions in the initial stages of crystallization and could be further explored in future work.

Overall, these results demonstrate that although the PP membrane does undergo measurable interfacial reorganization upon exposure to hypersaline LiF, its morphological and electrostatic characteristics remain sufficiently stable within the early simulation window.

## 3. Materials and Methods 

### 3.1. Membrane Preparation

Computational fluid dynamics (CFD) simulations were carried out on two different architectures: pristine dense membranes and dense membranes extracted at various times (0 ns, 150 ns, 300 ns) from molecular dynamics simulations, describing the MCr process of lithium salts. The procedure used for creating the membrane model is reported in detail by Prenesti et al. [22].

An isotactic PP chain of 300 monomers was generated. The modelling was conducted using the Material Studio 8.0 (BIOVIA) software, a specialized tool for atomistic and molecular-level modelling and simulation.

The generated chain was subsequently inserted into a confined amorphous layer with an average density of 0.85 g/cm^3^ at a temperature of 300 K. To ensure the sampling of the largest possible number of conformations, a simulation cell with smaller dimensions than the desired one (50 Å × 50 Å × 30 Å) was initially used, and subsequently, this was replicated along the three directions of *x*, *y*, and *z*, creating a supercell. During the model construction, argon was also used as a spacer to avoid the accumulation of monomers in high-density areas of the cell and to ensure a homogeneous distribution of the polymer.

Finally, graphene layers were utilized to guarantee the stabilization of the polymer surfaces and to create virtual ‘boundaries’ of the modelled structure. The confinement imposed on the PP membrane limits the excessive fluctuation of the chains, facilitating the geometry optimization procedure.

After constructing the polymeric membrane, a supersaturated lithium fluoride solution was positioned above the membrane interface. The combined system was then energy-minimized and equilibrated under NVT and NPT conditions for 100 ps each. The resulting equilibrated configurations were used as starting points for the production runs, which were performed in GROMACS 5.1.4 for 300 ns under NPT conditions

The selection of this time window is supported by previous MD studies on lithium fluoride crystallization and membrane interactions [22], which show that critical structural changes typically occur within the first 300 ns of simulation. Beyond this range, the system generally reaches a quasi-equilibrium state, where no significant evolution in either crystallization or polymer chain arrangement is observed.

The force field *oplsaa* was used to model the whole system, and *SPC/E* was the chosen model for water. Lennard Jones parameters are reported in Table 1.

Specifically, simulations were run at two different salt concentrations (5.8 M and 8.9 M), and for each condition, three temperatures were simulated. Each simulation was repeated twice for statistics. The initial configuration of the polypropylene (PP) membrane prior to contact with the ionic solution was used as the pristine (or virgin) membrane reference. This unperturbed structure served as the control system throughout the study. All subsequent analyses performed at varying salt concentrations and temperatures were systematically compared against this pristine membrane in order to assess any relative morphological or electrostatic changes induced by LiF crystallization conditions. PP structures were extracted in pdb format at 0 ns, 150 ns and 300 ns. These snapshots capture the evolution of the system from the moment of initial contact with the ionic solution (0 ns), through an intermediate state (150 ns), to a relaxed configuration after short-term equilibration (300 ns).

**Figure 9:**
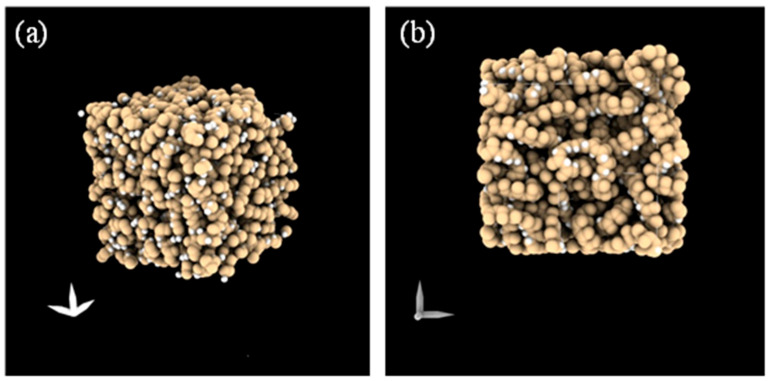
(**a**) Three-dimensional view of membrane structure; (**b**) *x*-*y* plane view of membrane structure.

They were therefore evaluated in terms of deviation from this baseline configuration. Structures were then converted to stl format with pyMol 3.1 (Schrödinger, New York, NY, USA) (Figure 9).

### 3.2. CFD Simulations Details and Mathematical Approach

After extracting the desired PP structures, the appropriate physical models were applied. To clarify the computational approach adopted in this work, we briefly summarize below the key aspects of our previous study. This approach involves performing CFD simulations with OpenFOAM under pure diffusion conditions along the three principal directions—*x*, *y*, and *z*—of a parallelepiped unit cell.

The first step of the present methodology focuses on the determination of the average effective diffusivity tensor (AEDT)—a conceptual tool used to recognize what may constitute the preferred diffusional paths within the membrane structure under investigation. As in the case of the local binary diffusivity tensor, we start from the constitutive equation describing the flow of a species in a three-dimensional space under purely diffusive conditions (Equation (1)).

(1) J¯T,xJ¯T,yJ¯T,z=−D¯e,xxD¯e,xyD¯e,xzD¯e,yxD¯e,yyD¯e,yzD¯e,zxD¯e,zyD¯e,zz·∆C∆x∆C∆y∆C∆z⇔J¯¯T=−D¯¯e·∆C

From this relationship, the concentration gradient is explicitly derived so that it can be expressed as a function of flux and diffusive resistivity (Equation (2)). Then, it is possible to proceed to determine all components of the resistivity tensor and, by inverting this tensor, to obtain the average effective diffusivity tensor (Equation (3)).

(2) ∆C∆x=−R¯e,xx JT,x−R¯e,xy JT,y−R¯e,xz  JT,z∆C∆y=−R¯e,yx JT,x−R¯e,yy  JT,y−R¯e,yz  JT,z∆C∆z=−R¯e,zx JT,x−R¯e,zy  JT,y−R¯e,zz  JT,z

(3) $${\overset{¯}{\underset{=}{D}}}_{e}=-{{\underline{\overset{¯}{\underset{¯}{R}}}}_{e}}^{-1}$$

The individual resistivity coefficients are calculated through the execution of CFD simulations with two kinds of appropriate boundary conditions (Equation (4)). These conditions are essential to ensure a purely diffusive and predictable flow condition at each point of the spatial domain. The boundary conditions implemented are as follows:

A constant, non-zero concentration gradient imposed between the two opposite faces of the computational domain of interest (*C*_(*a*,0) and *C*_(*a*,*L*)), after having designated a specific direction as preferential (e.g., the *x*-axis).

A zero-flux condition along the remaining directions (in this case, *y* and *z*) so that the flux lines have a unidirectional pattern, oriented counter to the imposed concentration gradient along the selected direction (from the surface with the highest concentration to the surface with the lowest concentration).

(4) Cx=xa=Ca,xCx=xb=Cb,x⇒∆C∆x,JT,x≠0JT,yy=ya=JT,yy=yb=0JT,yz=za=JT,yz=zb=0⇒ zero flux conditions 

(5) ∆C∆xJ¯T,y=J¯T,z=0=−R¯e,xx J¯T,x∆C∆yJ¯T,y=J¯T,z=0=−R¯e,yx J¯T,x∆C∆zJ¯T,y=J¯T,z=0=−R¯e,zx J¯T,x⇒R¯e,xx=−∆C∆xJ¯T,y=J¯T,z=0J¯T,xR¯e,yx=−∆C∆yJ¯T,y=J¯T,z=0J¯T,xR¯e,zx=−∆C∆zJ¯T,y=J¯T,z=0J¯T,x

Based on this scheme, three simulations (one for each main direction) are required to calculate all nine components of the resistivity tensor (3 × 3 matrix). The next step, once the mean effective diffusivity tensor is known, is to determine its diagonal form (Equation (6)), which is obtained by calculating the eigenvalues (${{\bar{D}}_{e,i}}^{\left(d\right)}$) and eigenvectors (${\underline{v}}_{n,i}$) of the tensor. The eigenvectors define a coordinate system along which the diffusion occurs independently, while the eigenvalues represent the components of the diagonal tensor.

(6) De¯¯d=Dde,1Dde,2Dde,3,    Dde,1 ⇔ vn,1Dde,2 ⇔ vn,2Dde,3 ⇔ vn,3

Once the average diagonal effective diffusivity tensor is known, the diagonal tortuosity tensor is determined (${\underset{=}{\tau }}^{\left(d\right)}$), whose components (eigenvalues) provide quantitative indications of the extent of preferential diffusion profiles. It is calculated using the inverse relationship between the diagonal effective diffusivity tensor, free-space diffusivity and porosity (Equation (7)).

(7) $${{\overset{¯}{\underset{¯}{\underset{¯}{D}}}}_{e}}^{\left(d\right)}=D\cdot \varepsilon \;\cdot {{\underset{=}{\tau }}^{\left(d\right)}}^{-1}$$

The last tensor quantity to be derived is the connectivity tensor ($\underline{\underline{\varphi }}$), which represents the amount of connection along each direction of the membrane. Several definitions are reported in the literature for the calculation of this quantity; in the present study, we used a relationship developed by our research group [30], according to which the connectivity factor φ is obtained as the inverse of the diffusional tortuosity (τ) (Equation (8)).

(8) $${\underline{\underset{¯}{\varphi }}}^{\left(d\right)}\equiv \;{\underset{=}{\tau }}^{\left(d\right)-1}\;\;\;\Leftrightarrow \;\;\;\left[\begin{array}{ccc} {\varphi_{11}}^{\left(d\right)} & & \\ & {\varphi_{22}}^{\left(d\right)} & \\ & & {\varphi_{33}}^{\left(d\right)} \end{array}\right]={\left[\begin{array}{ccc} {\tau_{11}}^{\left(d\right)} & & \\ & {\tau_{22}}^{\left(d\right)} & \\ & & {\tau_{33}}^{\left(d\right)} \end{array}\right]}^{-1}$$

The coefficients of the connectivity tensor will eventually be used to modulate the normalized eigenvectors, so as to ‘weight’ the preferred diffusion directions according to the degree of connections present along each direction (Equation (9)).

(9) $${\underset{¯}{u}}_{i}=\varphi_{i\;}\;{\underset{¯}{v}}_{n,i}$$

Within the methodology framework, both tortuosity and connectivity were adopted as they are able to offer an intuitive, conceptually lucid and meaningful representation of diffusion within porous media (porosity defined as the void volume between the polymeric chains).

Tortuosity (τ) is defined across a spectrum ranging from one (representing a relatively straight, preferential path) to infinity (a scenario where no connection exists between two discrete points of the domain), while connectivity (φ) (inverse of tortuosity) varies between a maximum of one (maximum degree of connection) and zero (no connection). Hence, the more tortuous the structure, the less the connection between two voids [31,32].

The procedure concludes with the calculation of three quantities:

- Overall tortuosity, calculated as the norm of the diagonal tortuosity tensor (Equation (10)).

(10) $$\tau_{ov}=\sqrt{{{\tau_{11}}^{\left(d\right)}}^{2}+{{\tau_{22}}^{\left(d\right)}}^{2}+{{\tau_{33}}^{\left(d\right)}}^{2}}$$

- Overall connectivity coefficient, calculated as the norm of the connectivity tensor (Equation (11)).

(11) $$\varphi_{ov}=\sqrt{{{\varphi_{11}}^{\left(d\right)}}^{2}+{{\varphi_{22}}^{\left(d\right)}}^{2}+{{\varphi_{33}}^{\left(d\right)}}^{2}}$$

- Anisotropy coefficient (Equation (12)).

(12) α=16φ11d−φ¯2 +φ22d−φ¯2 +φ33d−φ¯2 2 φ   

(13) $$where\;\;\;\bar{\varphi }=\frac{{\varphi_{11}}^{\left(d\right)}+{\varphi_{22}}^{\left(d\right)}+{\varphi_{33}}^{\left(d\right)}}{3}$$

### 3.3. Computational Mesh

The numerical representation of the geometry of the system under consideration is a fundamentally important step, since an accurate design guarantees both the accuracy of the results obtained and the computational efficiency of the process [31,32].

In the present study, the structure used allows for the interpenetration of the particles that make up the PP chains. This peculiar internal morphology is responsible for the creation of extremely narrow channels in certain portions of the material, within which diffusion occurs with greater difficulty and high concentration gradients occur. It is important that such regions are discretized using a larger number of computational grid elements in order to capture concentration variations accurately.

**Figure 10:**
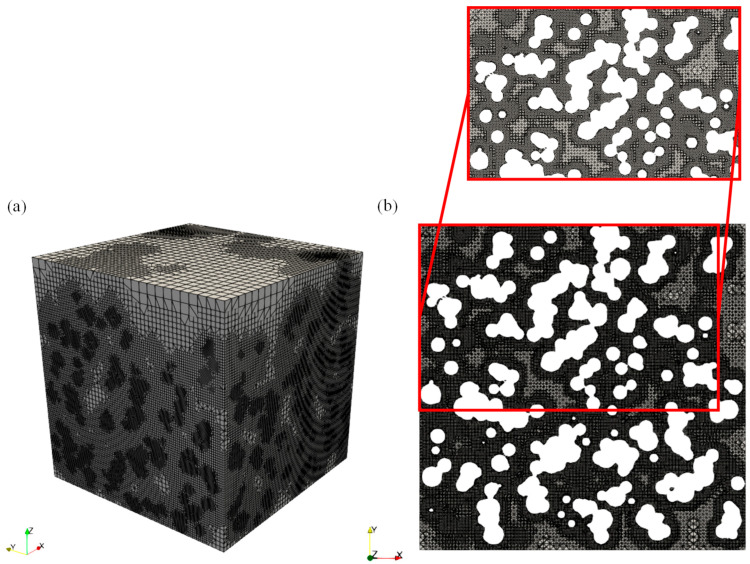
(**a**) Mesh on the whole structure; (**b**) Mesh on *x*-*y* plane with the relative zoom.

Consequently, various approaches may be adopted to account for this. The methodology used in the present study consisted of increasing the density of the computational grid in the channels that are narrower, seeking to optimize the accuracy of the simulations without unduly compromising the overall computational load of the structure. An example of the used mesh is reported in Figure 10.

Furthermore, the approach used aimed to ensure the independence of the results from the grid, since the results obtained must not vary significantly as the discretization changes. A pronounced sensitivity of the results to small changes in the grid would indicate insufficient density and, consequently, poor reliability.

Previous studies conducted by our research group already confirmed the validity of this procedure [31,32]. This indicates that making the computational grid narrower appears to be an effective technique to build a mesh when dealing with pure-diffusion systems.

### 3.4. Electrostatic Charge Distribution

The charge distribution on the membrane was performed by assigning the appropriate partial charge to each atom (Table 2). Charges are expressed as fractions of the elementary charge (*e*). Specifically, this procedure was carried out with ChimeraX 1.19.

**Figure 11:**
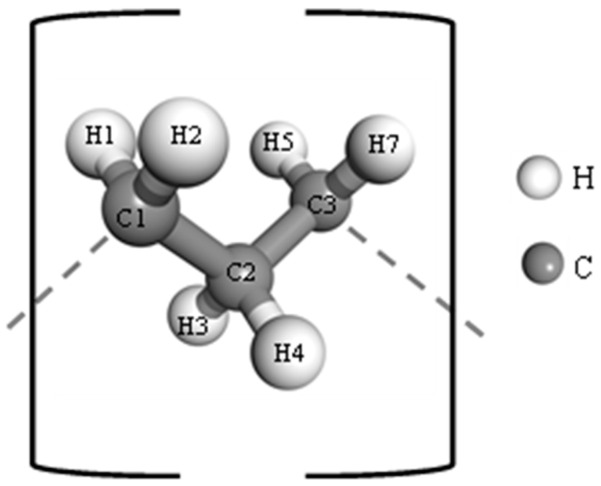
Polypropylene monomer.

Atom labels follow the scheme shown in Figure 11, with H7 referring to the terminal hydrogen, which appears only in the first and last monomer units of the polymer chain.

## 4. Conclusions

This study addressed the short-term morphological evolution of a dense isotactic polypropylene (PP) membrane model upon contact with highly concentrated LiF aqueous solutions under controlled thermodynamic conditions. The investigation was motivated by the increasing interest in understanding how solid–liquid interfacial interactions may influence membrane structure and performance during crystallization processes involving highly supersaturated systems. While experimental studies have highlighted the susceptibility of polymeric membranes to structural changes under such conditions, the molecular-level mechanisms involved remain largely unexplored.

To bridge this gap, we employed a multiscale computational strategy that integrates atomistic molecular dynamics (MD) simulations with an innovative computational fluid dynamic procedure (CFD). Developed within our research group, it was applied to different structural architectures extracted at successive times (0 ns, 150 ns, and 300 ns).

The adopted model intentionally neglects membrane porosity, ion transport, fluid permeation, or chemical degradation mechanisms. Instead, it focuses exclusively on probing local morphological descriptors in a dense, static membrane system over short simulation times (up to 300 ns). This approach allows for isolating and characterizing subtle physical rearrangements induced by temperature and solute concentration at the polymer–solution interface.

Our results demonstrate that global morphological descriptors such as tortuosity, connectivity, and void fraction remain essentially unchanged across all simulated conditions: temperature range (300–353 K) and LiF concentrations (5.8 M and 8.9 M). These observations suggest that the bulk structure of the PP matrix remains topologically stable in the early stages of exposure to hypersaline environments. Notably, no progressive trends were observed across the 300 ns time window, further supporting the conclusion that short-term interfacial interactions do not significantly disrupt the membrane’s internal architecture.

However, local structural rearrangements were observed through variations in anisotropy (α) and deviatoric anisotropy (αΔ), indicating that the polymer chains undergo non-negligible conformational adjustments when immersed in the ionic solution. These changes were more pronounced at lower temperatures and lower LiF concentrations, suggesting that thermal energy and electrostatic interactions modulate the degree of chain mobility. Although anisotropy values partially recovered toward the end of the simulation, they did not return to the levels of the pristine membrane, implying that the observed rearrangements are not fully reversible on the simulated timescale and result in a modest but detectable compaction and alignment of polymer chains.

The analysis of electrostatic surface charge distributions revealed additional effects of ion–polymer interaction. At a 5.8 M concentration, we observed a redistribution of partial atomic charges on the polymer surface, consistent with chain reorganization and exposure of terminal groups. This effect was attenuated at higher temperatures, likely due to the increased kinetic energy of the system. In contrast, simulations at 8.9 M displayed minimal variation in surface charge, pointing to the dominant role of strong Coulombic interactions in restricting chain mobility and preserving surface structure. Across all conditions, the PP surface retained a net negative character, consistent with experimental zeta potential values and relevant for understanding possible influences on heterogeneous nucleation.

In conclusion, this work presents a computational investigation focused on the initial physical interactions between a dense PP membrane and a hypersaline solution of LiF. Within the boundaries of the applied model, which excludes fluid transport and degradation phenomena, this study confirms that polypropylene preserves its overall morphological framework in the early stages of exposure. At the same time, it highlights measurable local reorganizations of chain orientation and electrostatic properties that may influence subsequent interfacial processes. These findings do not imply long-term structural stability but rather contribute to clarifying the short-timescale morphological response of the material, offering a computational framework that may support more complex future simulations and experimental comparisons aimed at optimizing membrane design for crystallization and separation technologies.

## Figures and Tables

**Table 1:** Lennard-Jones (LJ) parameters.

Specie	σ, nm	ε, kJ mol^−1^	Water Model
Li^+^	0.212645	0.0764793	SPC/E
F^−^	0.273295	3.01248	
C (PP)	0.3500	0.276144	
H (PP)	0.2500	0.125520	

**Table 2:** Partial atomic charges for polypropylene monomer units in the OPLS-AA force field.

Atoms	Charge
C1	−0.053
C2	−0.159
C3	−0.106
H1, H2, H3, H4, H5, H6, H7	0.053

## Data Availability

The original contributions presented in this study are included in the article. Further inquiries can be directed to the corresponding authors.
